# Efficiency and factors influencing efficiency of Community Health Strategy in providing Maternal and Child Health services in Mwingi District, Kenya: an expert opinion perspective

**DOI:** 10.11604/pamj.2015.20.88.4711

**Published:** 2015-01-30

**Authors:** Japheth Mativo Nzioki, Rosebella Ogutu Onyango, James Herbert Ombaka

**Affiliations:** 1 Department of Environmental Health, University of Kabianga, Kericho, Kenya; 2 Department of Public Health, Maseno University, Kisumu, Kenya; 3 Department of Biomedical Sciences and Technology, Maseno University, Kisumu, Kenya

**Keywords:** Community Health Strategy, efficiency, factors influencing efficiency, aternal and Child Health, Mwingi district

## Abstract

**Introduction:**

Community Health Strategy (CHS) is a new Primary Health Care (PHC) model in Kenya, designed to provide PHC services in Kenya. In 2011, CHS was initiated in Mwingi district as one of the components of APHIA *plus kamili* program. The objectives of this study was to evaluate the efficiency of the CHS in providing MCH services in Mwingi district and to establish the factors influencing efficiency of the CHS in providing MCH services in the district.

**Methods:**

This was a qualitative study. Fifteen Key informants were sampled from key stakeholders. Sampling was done using purposive and maximum variation sampling methods. Semi-structured in-depth interviews were used for data collection. Data was managed and analyzed using NVIVO. Framework analysis and quasi statistics were used in data analysis.

**Results:**

Expert opinion data indicated that the CHS was efficient in providing MCH services. Factors influencing efficiency of the CHS in provision of MCH services were: challenges facing Community Health Workers (CHWs), Social cultural and economic factors influencing MCH in the district, and motivation among CHWs.

**Conclusion:**

Though CHS was found to be efficient in providing MCH services, this was an expert opinion perspective, a quantitative Cost Effectiveness Analysis (CEA) to confirm these findings is recommended. To improve efficiency of the CHS in the district, challenges facing CHWs and Social cultural and economic factors that influence efficiency of the CHS in the district need to be addressed.

## Introduction

Community Health Strategy (CHS) is a new Community Health Worker (CHW) led Primary Health Care (PHC) intervention in Kenya. The CHS intends to improve the health status of Kenyan communities through building the capacity of the Community Health Workers (CHWs) to provide PHC services, strengthening health facility'community linkages and strengthening the community to progressively realize their rights to access quality healthcare and to seek accountability from health facility based services [1]. CHS was designed in 2006 to support the delivery of Kenya Essential Package for Health (KEPH) at level one (Community level). The CHS program structure provides for creation of Community Units (CUs) as the basis of PHC service provision. Each CU is supposed to serve approximately 5000 people. The service providers in the CUs are well trained Community Health Workers (CHWs) and Community Health Extension Workers (CHEWs). CHWs are members of the community identified by the community and trained to serve the same communities they come from while CHEWs are trained health professionals (Nurses and Public health officers trained at certificate and/or diploma levels and working for the Ministry of Health). Each CHW is required to provide PHC services to 20 households. The responsibilities of CHEWs are; to supervise CHWs (each CHEW is required to supervise up to 25 CHWs), to facilitate trainings in the community and to provide a linkage between CHWs and health facility [1].

Since inception of the CHS in the year 2007, the Government of Kenya (GoK) guided by the 2008-2012 Ministry of Public Health and Sanitation (MoPHS) Strategic Plan and with the help of development partners initiated implementation of CHS pilot programs in various districts in the Country [2]. In 2011 the United States Agency for International Development (USAID) funded the AIDS, Population and Health Integrated Assistance *plus kamili* (APHIA *plus Kamili*) program for zone 4 for five years starting 2011 and ending 2015. Zone 4 represented both Eastern and Central Kenya. In the same year Community Health Strategy (CHS), one of the components of APHIA *plus kamili* program was initiated in Mwingi district by the Ministry of Public Health and Sanitation (MoPHS) in partnership with the African Medical and Research Foundation (AMREF) [3]. Evaluating a health care program's efficiency and identifying the factors that influence efficiency of health care programs provides an opportunity to improve the efficiency of such programs by Increasing service provision using existing resources [4]. Besides optimum utilizing of resources, efficient PHC programs have been found to contribute to the effectiveness of overall health service provision [5]. CHS is not only one of the Community led health initiatives driving Kenya into realizing Millennium Development Goals (MDGs) number 4 and 5 but is also a component in the health flagship projects of Kenya's vision 2030 [6]. Since inception of the program in Mwingi district no study has been conducted to evaluate the efficiency of the program in providing Maternal and Child Health (MCH) services in the district. Similarly the factors influencing efficiency of the CHS in providing MCH services are not known. This underscores the importance of evaluating efficiency of the CHS program as well as establishing the factors influencing efficiency of the same program in providing MCH services in Mwingi district. The aim of this study was to evaluate the efficiency of the CHS in providing MCH services in Mwingi district and identify the factors influencing efficiency of the CHS program in providing MCH services in the same district.

## Methods

### Study location

This study was conducted in Mwingi district. It was part of a larger study conducted to evaluate the effectiveness of CHS in providing MCH services in Mwingi district. In the larger study data was collected from a total of 2532 households in both Mwingi district (study site) and Kyuso district (control site). Mwingi district is semi-arid and has a total population of 227, 878 people (107,186 Male and 120,692 Female) and 45,445 households and covers an area of 5,217.1 Square Kilometers [7]. The district has a population growth rate of 2.4%, crude birth rate of 47.6 per 1000, crude death rate of 11.3 per 1000, infant mortality rate of 82 per 1000, neo-natal mortality rate of 38 per 1000, post neo-natal mortality rate of 30.2 per 1000, under five mortality rate of 120 per 1000, total fertility rate of 5.0 and a life expectancy of 55 years [8].

### Study participant's characteristics

The study participants were key informants drawn from stakeholders of the CHS program. Three groups were identified from the stakeholders on the basis of their knowledge on efficiency and knowledge on factors influencing efficiency of the CHS program. These groups are; health experts, people implementing the program and people utilizing the services of the program.

### Sampling procedure and selection of key informants

Two sampling procedures were employed namely; purposive and maximum variation sampling. Purposive sampling was applied in selecting the sampling frame in this study which was the stakeholders of the CHS Program. Maximum variation sampling was applied to select key informants from the sampling frame. The aim was to increase representation of expert opinions on efficiency and factors influencing efficiency of the CHS program. Three groups of stakeholders namely; health experts, people implementing the program and people utilizing the services of the program were identified. In the three groups five categories of stakeholders were sampled namely; public Health Officers, Clinicians, managers coordinating the CHS program, Community Health Workers (CHWs) implementing the CHS, and community members utilizing services of the program. Three key informants were sampled from each category. Total number of participants (N) was 15. However, there was provision to increase this number in the event that new issues emerged by the time of interviewing the third participant in each category.

### Data collection approach and data collection tools

Semi structured interviews were used for data collection. In-depth interview question guides were used in data collection. Table 1 represents the in-depth interview question guide. The questions represented were the core questions for the interviews. However more questions were introduced in the course of the interviews to probe issues for clarity. A pilot study was conducted by the first author to help refine the key informant question guide.

**Table 1:** Key informant Interview question guide used in evaluating efficiency and factors influencing efficiency of Community Health Strategy (CHS) in Providing MCH Services in Mwingi District; Kenya

Q1.	APHIA *plus Kamili* /AMREF/MoPHS initiated the CHS three years ago in this district with the aim of improving MCH. Which resources/inputs have been employed in the CHS program
Q2.	Since inception of the CHS program in January 2011, have you observed any outcomes on MCH that can be attributed with the program activities? If yes name them.
Q3.	Do you think the program outcomes have been produced with optimal/minimum use of resources? Whether ‘Yes’ or ‘No’ or ‘not sure’ Give an explanation
Q4.	What do you consider as the key things that hinder/have hindered efficiency of the CHS in providing MCH services?
Q5.	What do you consider as key to promoting Efficiency of the CHS in providing MCH service?

### Data collection process

After meeting all procedures of research ethics, fifteen in-depth interviews were conducted from different locations in Mwingi depending on the location of the Key informant. Data was collected using both hand written notes and audio taped materials. Respondent validation of data was conducted by giving the interviewees chance to review the written scripts before preparing the final transcripts. In the five categories of stakeholders, the interviews went on until saturation in that the third interviewee in each category was constantly repeating the issues expressed by the first and second interviewees. Therefore the total number of respondents remained fifteen (N=15).

### Data management and analysis

Data management and analysis was done using a qualitative data management and analysis software-the NVIVO (version 10). Two analysis techniques namely; framework analysis and use of quasi statistics were employed. Data was analyzed using the following steps; data familiarization, identifying a thematic framework, indexing, charting, interpretation and application of quasi statistics. Familiarization with data was done by listening to audio data tapes and reading the field notes. Data was then classified and summarized into a thematic framework. A coding scheme (referred to as “Nodes” in the NVIVO 10) was then developed through a process of indexing with the themes identified becoming the labels for the codes. Following this was rearranging the data according to thematic content in a chart in a format that enabled participants and their original responses to be viewed against the themes created. Deliberate effort was made to preserve the integrity of individual accounts/responses in the entire process. The next step was interpretation of data which involved comparing the narratives within and between the themes. Following this was application of quasi statistics which involved creating a summary in each theme of the number and percentage of respondents who supported a theme, had a dissenting opinion against a theme and these who neither supported nor opposed a certain theme.

### Ethical consideration

The National Council of Science and Technology (NCST) of the Government of Kenya (GoK) conducted an ethical review of the study and approved it. Written informed consent was obtained from all the study participants before the study commenced.

## Results

The findings of this study have been classified into two categories: one; expert opinions on the efficiency of the Community Health Strategy (CHS) in providing Maternal and Child Health(MCH) services in Mwingi district and two; factors influencing efficiency of the CHS in providing MCH services in the same district.

### Expert opinions on the efficiency of the CHS in providing MCH services in Mwingi district

Results on efficiency of the CHS clustered on one theme; that is the program provided MCH services efficiently. Data collected from the 15 key informants on whether minimum resources were spent to produce maximum MCH outcomes indicated that the CHS program was efficient in providing MCH services with 100% support from the respondents. Six key informants involved in the implementation of the CHS (three CHS program managers and three CHWs) acknowledged that most activities that were scheduled to be implemented were done against a backdrop of limited resources. Volunteer CHWs were trained in time, formation and mapping of Community Units (CUs) was done and the CHWs started rolling out MCH services prescribed in the CHS in time. It was however noted that, provision of essential drugs as well as provision of Family Planning (FP) services has not been rolled out due to lack of essential drugs, FP drugs and condoms in the CHWs kits.

*“Volunteer CHWs are now providing MCH services in their CUs… the impact of their work has been felt not only in the community but also in our health centers…we have more women attending ANC services and delivering in medical facilities under the care of a skilled health worker than before…and the resources we are using are minimal…it is cost effective to work with volunteers”, (Key informant, 02)*.

Six key informants from the healthcare service provision sector (Clinicians and Community Health Nurses) also acknowledged that the CHS is the single program that has transformed the lives of women and children in the district using the least resources possible. They admitted having observed a positive change in MCH outcomes in the district.

*“The idea of using a motivated team of volunteer CHWs to improve the health of mothers and children is the best thing that has happened in the lives of women and children in Mwingi district…The change in their health is real…we have observed an increase in number of expectant women utilizing ANC services, an increase in number of expectant women delivering in medical facilities, and a decrease in the cases of maternal and child health problems in medical facilities…. amount of resources used is minimal” (key informant, 8)*.

Three key informants from the community were also for the opinion that the CHS was utilizing available resources efficiently to produce desired MCH outcomes. They acknowledged having observed a positive change in the health of mothers and children at the household level with minimal expenditure of resources by CHWs.

*The CHWs have done a recommendable job in the Community….. Our women and children are healthier than before…. This is the only program I have observed commendable results with minimal use of resources“ (Key informant, 14)*.

### Factors influencing efficiency of the CHS in providing MCH services in Mwingi district

Opinions on the factors that have influenced the efficiency of the CHS in providing MCH services in Mwingi district clustered around three themes namely; challenges facing CHWs, social cultural and economic factors and motivation of volunteer CHWs.

### Challenges facing CHWs in the implementation of the CHS program

Eighty percent (12/15) of the respondents identified challenges facing CHWs as one of the factors that slowed the progress of CHWs in attaining their goals in their respective CUs. This in-turn slowed their productivity hence impeding efficiency of the CHS in providing MCH services. Among the key challenges identified were; lack of facilitation in their movement, poverty, lack of support from their spouses, delayed payment of volunteer allowances, overworking and incomplete CHWs kits.

*“Many CHWs are poor…. they are forced to strike a balance between struggling to make a living and volunteering in the CHS program,…. As a result many CHWs have opted out of the CHS program…, (Key informant, 04). “The terrain in the district is expansive with some households in areas that are remote and difficult to reach. …accessing such households without facilitation has been a major challenge…, (Key informant, 07). “Some CHWs have had difficulties in convincing their spouses for support…their spouses prefer a paying job as opposed to volunteering”, (Key informant, 06). “We are not providing some of the services we are supposed to provide to the community simply because we are not facilitated…we do not have family planning pills. Condoms, oral rehydration salts, and essential drugs in our kits”, (Key informant, 10). “Our volunteer allowance is only Kshs.500 paid any time we have a meeting to return our reports. The money is hardly paid in time… this has demotivated many CHWs and as a result they have dropped out of the program”, (Key informant, 5). “Some of us have up to 600 households in our CUs..The work is just too much for an individual”, (Key informant, 4)*.

### Social cultural and economic factors that influence efficiency of the CHS in Mwingi District

Seventy three percent (11/15) of the study respondents identified key social cultural and economic factors which influenced efficiency of the CHS in providing MCH services negatively. Some factors identified influenced efficiency by slowing down the uptake of MCH services in the community while other factors influenced efficiency by creating an environment which does not enable MCH services provided by CHWs to thrive. These factors are; religion, Female Genital Mutilation (FGM), poverty, food insecurity, and various constrains in health care service provision which include; understaffing, insufficient emergency obstetric care services and insufficient medical supplies (drugs and equipment).

*“Some religious families especially the believers of the Kavonokya faith do not allow CHWs to access their households…. they don't seek any services from hospitals or CHWs…their convictions do not allow them to utilize health care services” (Key informant”, 011). “The main cause of malnutrition in this community is poverty and food insecurity. ..the CHS program cannot change the situation….. This creates an environment that does not support the efforts CHWs put in place to improve MCH in this district”, (Key informant, 010) “General health service provision in the district is poorly resourced… We are understaffed, we do not have sufficient medical supplies to serve the increasing number of clients……most of our ambulances are grounded…we cannot respond to emergency obstetric needs in the community promptly. These issues do not compliment the efforts of CHWs in the CHS program,” (key informant, 06)*.

### Motivated team of CHWs

Sixty seven percent (10/15) of the respondents identified motivation of CHWs as the driving force behind the efficient implementation of the CHS program. This according to the respondents is the force behind the realization of improved MCH outcomes with minimum expenditure in resources.

*“It's true we have had our challenges in this work…it's true the challenges have made a good number of the CHWs drop out of the team…. but even so…. the team that has endured this challenges is the winning team…they are highly motivated…their driving force is to see a positive change in the health of women and children in the district.,” (Key informant, 06)*.

## Discussion

### Efficiency of community health workers’ led interventions in provision of MCH services

Expert opinions indicate that the CHS is efficient in providing MCH services in Mwingi district. Studies have shown that community led Health programs providing MCH services in resource poor settings like the CHS have been implemented with great efficiency in many parts of the world. The Millennium Villages Project is one of the projects which has used Community Health Workers to improve MCH outcomes efficiently [9]. Through the work of a widely deployed team of CHWs providing integrated MCH services in all households, Bangladesh was praised as an example of “good health at low cost” and has been recognized by United Nations for its exemplary progress towards achievement of Millennium Development Goal (MDG) four and five [10]. Similarly, a study in India conducted to analyze the cost effectiveness of Primary Health Care (PHC) services delivered through CHWs in 3 North Indian States concluded that CHWs offered efficient low-cost option to deliver PHC services [11].

Though expert opinions in this study indicate that the CHS is efficient in providing MCH services and though previous studies do support the findings of this study, an economic analysis to establish the cost effectiveness of the CHS in providing MCH services in Mwingi district is recommended. This recommendation is based on two things; one the fact that the validity and reliability of a Cost effectiveness Analysis (CEA) of an intervention is superior compared to an expert opinion analysis because it will quantify all costs including CHWs time used in the interventions against the effects of the intervention and therefore it will provide strong scientific evidence that can inform policy and two, this study identified many factors that influenced efficiency of the CHS negatively and therefore a quantitative economic survey to confirm these findings is recommended

### Factors influencing efficiency of community health workers’ led interventions in provision of MCH services

This study established the factors influencing efficiency of the CHS in providing MCH services in Mwingi district as; challenges facing CHWs, social cultural and economic factors and motivation of volunteer CHWs. Regarding challenges facing CHWs, previous studies have reported that CHWs face many challenges in implementing their programs. These challenges include; lack or shortage of materials/supplies, lack of Equipment necessary to cope with special weather conditions, lack of adequate transportation for hard-to-access areas, inconsistent remuneration, lack of performance-driven rewards, inadequate mentoring, supervision and support, lack of recognition, lack of opportunity for career advancement, lack of CHWs policy and in adequate community Participation [12–15]. However, studies linking the challenges CHWs face with efficiency of the programs they are implementing are scarce. A study conducted to establish ways of increasing CHW productivity recommends program managers to create enabling work environments for CHWs which encompasses four essential elements-manageable workload, supportive supervision, adequate supplies and equipment, and respect from the community and the health system [16]. It will be therefore valid to argue that challenges CHWs face in the course of their work hinder their performance and thus compromising their productivity. These challenges therefore are inefficiencies which if eliminated would increase productivity of CHWs without any increase in program resources. This in turn would increase efficiency of CHWs’ led interventions which have been regarded as “low cost-high impact options” that can be used to improve MCH outcomes in resource-constrained Countries [15, 17]. In Kenya the CHS, has been earmarked as one of the programs that will help the country achieve not only MDG 4 and MDG 5 but also the country's vision 2030 [18]. Though expert opinions do indicate that the CHS program is efficient, the challenges facing CHW present an opportunity to improve efficiency of the program. As indicated earlier in this paper, addressing these challenges will increase the productivity of CHWs without an increase in resources. Despite the CHS being one of the vision 2030 projects, Kenya does not have a CHW policy. Development of a policy guideline for CHWs by the Ministry of Health of the Government of Kenya is a step that could help solve the many challenges facing CHWs in the Country. In regard to social-cultural and economic factors that were found to influence efficiency of the CHS in providing MCH services, all the factors identified namely; religion, Female Genital Mutilation (FGM), poverty, food insecurity, and various constrains in health care service provision which include; understaffing, inadequate emergency obstetric care services and insufficient medical supplies (drugs and equipment) have also been identified as key determinants of MCH in various parts of the world [10, 19–24]. The findings of this study therefore indicate that social-cultural and economic factors that influence MCH also do influence efficiency of CHW led interventions aimed at improving MCH services to a great extent.

Though data linking social-cultural and economic determinants of MCH to efficiency of CHW led interventions is scarce, a recent study conducted in Tanzania established that efficiency of PHC services in the country depended on the number of staff in the health facilities, the population-staff ratio, and the quality of emergency obstetric care services provided [25]. This study indicates that social economic determinants of Health which the World Health Organization Commission on Social Determinants of Health refers to “the root causes of disease and health inequalities” [26] have capacity to influence efficiency CHW led health interventions. Though there is need for research to explore this phenomenon, addressing the social cultural and economic determinants of MCH in Mwingi district is required in order to improve efficiency of the CHS in providing MCH services in the district. This will help in creating an enabling environment for CHWs which in turn will increase their productivity. Regarding motivation of CHWs as a determinant of efficiency in Mwingi district, studies in Tanzania, Guatemala, Uganda, Nepal and India have shown that a highly motivated team of CHWs deliver services in an efficient manner while a low motivated team of CHWs will decrease the benefits of investments in CHW led interventions [27–30]. These findings are in line with the responses of expert opinions in Mwingi district which attribute efficiency of the CHS in providing MCH services in the district with a motivated team of CHWs. This study did not establish the factors motivating CHWs in the CHS program in Mwingi district and therefore an in-depth evaluation to establish factors that have influenced CHWs to provide MCH services efficiently against a plethora of challenges facing them is recommended.

## Conclusion

Though expert opinions in this study indicate that the CHS is efficient in providing MCH services, an economic analysis to establish the cost effectiveness of the CHS in providing MCH services in Mwingi district is recommended. This recommendation is based on two things; one the fact that the validity and reliability of a Cost effectiveness Analysis (CEA) of an intervention is superior compared to an expert opinion analysis and therefore it will provide strong scientific evidence that can inform policy and two, this study identified many factors that influenced efficiency of the CHS negatively and therefore a quantitative economic study to confirm these findings is recommended. The study established three factors that influence efficiency of the CHS program in Mwingi district namely; challenges facing CHW's in implementing the CHS program, Social cultural and economic factors influencing MCH in the district and motivation of the CHW's. The challenges facing CHWs act as inefficiencies which impend their productivity and addressing these challenges would improve this productivity with minimal or no increase in resources. We therefore recommend the Ministry of Health of the Government of Kenya to take measures (including development of a CHWs policy) to address these challenges. Similarly the Government of Kenya should employ efforts to address the social-cultural and economic determinants of MCH in Mwingi district which have also been found to influence the efficiency of the CHS. Lastly we recommend a study to identify factors motivating and demotivating the CHWs implementing the CHS program in Mwingi district. This may be used to inform the development of a friendly CHWs policy that would help improve the efficiency of their programs.
